# Enhancement of Immune Effector Functions by Modulating IgG’s Intrinsic Affinity for Target Antigen

**DOI:** 10.1371/journal.pone.0157788

**Published:** 2016-06-20

**Authors:** Yariv Mazor, Chunning Yang, M. Jack Borrok, Joanne Ayriss, Karen Aherne, Herren Wu, William F. Dall’Acqua

**Affiliations:** 1 Department of Antibody Discovery and Protein Engineering, MedImmune, Gaithersburg, Maryland, United States of America; 2 Department of Biopharmaceutical Development, MedImmune, Gaithersburg, Maryland, United States of America; National Cancer Institute, NIH, UNITED STATES

## Abstract

Antibody-mediated immune effector functions play an essential role in the anti-tumor efficacy of many therapeutic mAbs. While much of the effort to improve effector potency has focused on augmenting the interaction between the antibody-Fc and activating Fc-receptors expressed on immune cells, the role of antibody binding interactions with the target antigen remains poorly understood. We show that antibody intrinsic affinity to the target antigen clearly influences the extent and efficiency of Fc-mediated effector mechanisms, and report the pivotal role of antibody binding valence on the ability to regulate effector functions. More particularly, we used an array of affinity modulated variants of three different mAbs, anti-CD4, anti-EGFR and anti-HER2 against a panel of target cell lines expressing disparate levels of the target antigen. We found that at saturating antibody concentrations, IgG variants with moderate intrinsic affinities, similar to those generated by the natural humoral immune response, promoted superior effector functions compared to higher affinity antibodies. We hypothesize that at saturating concentrations, effector function correlates most directly with the amount of Fc bound to the cell surface. Thus, high affinity antibodies exhibiting slow off-rates are more likely to interact bivalently with the target cell, occupying two antigen sites with a single Fc. In contrast, antibodies with faster off-rates are likely to dissociate each binding arm more rapidly, resulting in a higher likelihood of monovalent binding. Monovalent binding may in turn increase target cell opsonization and lead to improved recruitment of effector cells. This unpredicted relationship between target affinity and effector function potency suggests a careful examination of antibody design and engineering for the development of next-generation immunotherapeutics.

## Introduction

Unconjugated monoclonal antibodies (mAbs) are now a mainstay for cancer therapy and represent the fastest growing class of biological therapeutics [1–4]. Many of these immunotherapeutics, upon binding to cell surface antigens, can engage Fcγ receptors (FcγR) expressed on immune effector cells or interact with complement 1q (C1q) and exert their biological activity through Fc-mediated effector mechanisms such as complement-dependent cytotoxicity (CDC), antibody-dependent cell-mediated cytotoxicity (ADCC) and antibody dependent cell-mediated phagocytosis (ADCP) [3, 5, 6]. The importance of the Fc–FcγR interaction is underlined by the clinical efficacy of several cornerstone antibody therapeutics, including: rituximab (anti-CD20), trastuzumab (anti-HER2) and cetuximab (anti-EGFR). Patients with allotypic polymorphism in FcγRIIIA were shown to display enhanced antibody binding and, as a result, improved immune response compared to patients with less reactive allotypes [7–13].

Over the past two decades substantial efforts have been invested in technologies that enhance antibody-mediated effector functions [14]. By employing glycoengineering and mutagenesis, these technologies predominantly focused on improving the affinity between the antibody Fc and activating FcγRs or to C1q [15–19]. However, while much has been reported on the cellular and molecular mechanisms that regulate Fc-mediated effector functions, including the significance of distinct FcγRs [20–22] and their interaction with different human IgG subclasses, [23–25] surprisingly very little is known about how antibody binding affinity to the target antigen affects effector function potency. To date, only one study investigated the relationship between antibody’s intrinsic affinity and ADCC potency [26]. In this study, Weiner and colleagues reported that affinity-improved variants of the anti-HER2 IgG C6.5 exhibited potentiated in-vitro ADCC compared to mutants with reduced affinity. Specifically, the high-affinity variant H3B1 (0.56 nM) was shown to mediate the greatest level of cell cytotoxicity against tumor cell lines with disparate levels of HER2 expression, followed by the moderate-affinity variant C6.5 (23 nM) and the low-affinity G98A (270 nM). The authors therefore concluded that the intrinsic antibody affinity for the target antigen clearly influences the extent and efficiency of ADCC, and that this correlation remains valid in tumor cell lines with widely disparate target antigen density.

We recently reported the development of an array of affinity-reduced variants of the anti-CD4 ibalizumab mAb by employing alanine mutagenesis to core contact residues in complementarity-determining region (CDR) H3 and L3 [27]. The IgG variants exhibited a ~2-100-fold reduction in affinity compared with the parental sequence. To better understand how antibody binding affinity to the target antigen affects effector function potency, in the current study, we assessed the capacity of several CD4 affinity-reduced IgG variants to mediate ADCC depletion of primary human CD4^+^ T cells isolated from healthy donors. Interestingly, we discovered that at saturating antibody concentrations, variants with reduced CD4 affinity exhibited superior ADCC. In particular, the level of cytotoxicity was inversely proportional to the reduced intrinsic affinity to CD4. Similar results were obtained when the IgG variants were tested for their ability to eradicate the target CD4^+^ T cells by other effector mechanisms such as CDC and ADCP. In an effort to understand the generality of our findings, we generated a series of affinity-reduced variants of the anti-EGFR GA201 mAb [28] and also reconstructed the anti-HER2 antibody sequences depicted by Tang et al and evaluated their capacity to elicit ADCC in vitro against various tumor cell lines expressing different levels of target antigen. We show for the first time that IgG antibodies with moderate intrinsic affinities, similar to those generated by the natural humoral immune response, promote enhanced effector functions compared to affinity-improved engineered antibodies. We further demonstrate how antibody binding valence to the target antigen regulates effector functions and provide a qualitative model to explain our results. These findings have significant implications for the development of clinically optimized mAbs that mediate effector functions.

## Results

### Affinity-reduced anti-CD4 IgG variants exhibit enhanced effector functions

**Fig 1:**
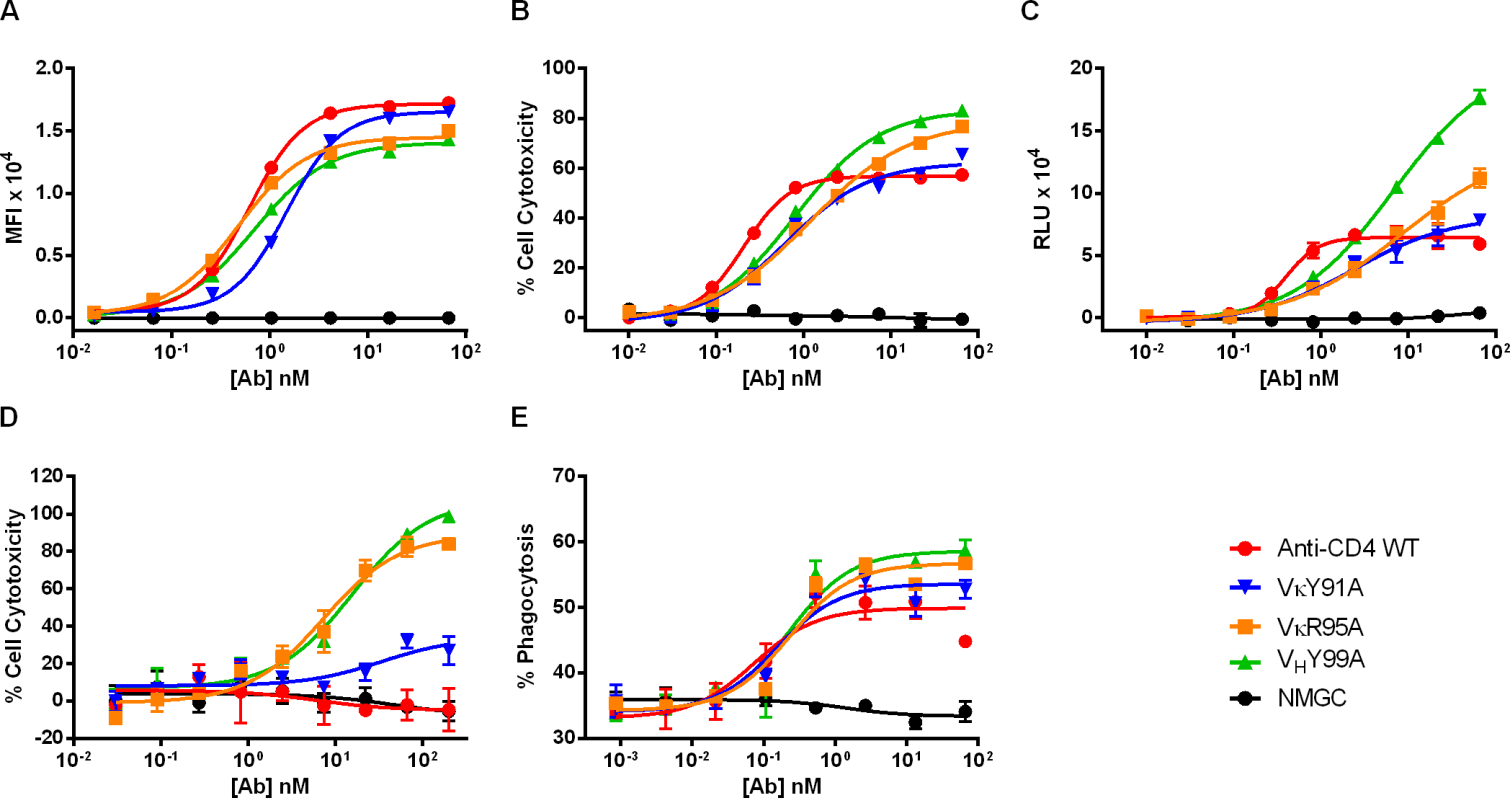
Cell binding and effector functions activity of anti-CD4 IgG variants. (A) Cell binding of anti-CD4 variants to CD4^+^ T cells. (B) ADCC activity of anti-CD4 variants against CD4^+^ T cells using KC1333 NK cells as effector cells. (C) Reporter-based ADCC activity of anti-CD4 variants against CD4^+^ T cells using NK92/NFAT cells as reporter cells. (D) CDC activity of anti-CD4 variants against CD4^+^ T cells using Rabbit complement at a final concentration of 11% (v/v). (E) ADCP activity of anti-CD4 variants against CD4^+^ T cells using human THP-1-derived macrophages as effector cells. NMGC represents isotype control antibody. Each point represents the mean values of triplicate wells and the standard deviation is represented by error bars. See (Table 1) for statistical analysis and *P* values.

Affinity-reduced variants of the anti-CD4 ibalizumab mAb were generated and produced as mammalian IgG as previously described [27]. All antibody preparations were rigorously purified to remove residual high molecular weight protein aggregates that may affect the sensitivity and integrity of effector function assays conducted in this study. Binding kinetics to CD4 were determined by Octet analysis (Table 1), and cellular binding properties to CD4^+^ T cells were determined by flow cytometry (Fig 1A). As shown, at saturating antibody concentrations variants with improved affinity to CD4 exhibited higher median fluorescence intensity (MFI) values compared to lower affinity variants. To determine the relationship between antigen binding affinity and the capacity to promote effector functions, we tested the ability of the IgG variants to mediate ADCC against CD4^+^ T cells isolated from healthy donors. The NK-derived cell line KC1333 were used as effector cells and cell cytotoxicity was determined by means of flow cytometry. As opposed to the cell binding results, at saturating antibody concentrations, the CD4 affinity-reduced variants mediated a greater degree of cytotoxicity compared with the parental ibalizumab IgG (Fig 1B). Particularly, the level of cytotoxicity was inversely proportional to the decreased intrinsic affinity to CD4. To faciliate comparative analysis of the data, we calculated the half maximal effective concentration (EC_50_) values and compared against the % cell cytotoxicity obtained at max antibody concentration used. As shown in Table 2, while the parental IgG exhibited a lower EC_50_ value compared with the values obtained for the affinity-reduced IgGs, at max antibody concentrations the affinity-reduced variants exhibited statistically significant superior cytotoxicity (*P* < 0.0001). This discrepancy between EC_50_ values and cytotoxicity at max antibody concentration may suggest that at limiting antibody concentrations, enhanced affinity accelerates target cells opsonization by rapidly achieving a threshold level of antibody-Fc domains required for recruitment of effector cells. Similar results were obtained using the previously described highly sensitive NK92/NFAT reporter ADCC assay [27]. As shown in Fig 1C, this assay measures effector cell signaling rather than target cell death. The results correlated well with the cytotoxic ADCC data and provided better resolution between tested antibodies. In an effort to understand whether this observation is unique to ADCC, we assessed the ability of the CD4 affinity-reduced variants to eradicate the target CD4^+^ T cells by additional immune mechanisms for example CDC and ADCP. For CDC analysis, antibodies were incubated with target cells in the presence of complement at a final concentration of 11% (v/v), and cell cytotoxicity was determined by imaging cytometry. For ADCP assessments, human macrophages were obtained by differentiation of THP-1cells using vitamin D-3, and cell phagocytosis was determined by means of flow cytometry. In agreement with the ADCC data, at higher antibody concentrations variants with reduced CD4 affinity exhibited potentiated CDC (*P* < 0.001)(Fig 1D) and ADCP activities (*P* < 0.003) (Fig 1E). The EC_50_ values and effector function potency at max antibody concentration are summarized in Table 2. The disparity between the cell binding signals observed at max antibody concentration and effector function potency could be explained by the different nature of the assays. In cell binding assays, following incubation of the antibodies with target cells the cells are subjected to two cycles of wash while the effector function assays depicted in this work are non-wash assays. It is therefore possible that affinity-reduced variants with weakened binding capabilities are washed out and hence the improved target cell opsonization suggested for the lower affinity variants cannot be determined in assays involving a washing step. In fact, when the ADCC activity of the anti-CD4 variants was assessed under washing conditions, at saturating antibody concentrations the low-affinity variants exhibited inferior cytotoxic activity compared to the high-affinity parental IgG (data not shown). These results confirm our assumption that affinity-reduced variants with weakened binding to the target antigen are more prone to be washed out from the cell surface and hence exhibit reduced capability to engage with effector cells.

**Table 1:** Binding affinity of IgG to target antigens.

		Intrinsic affinity			Apparent affinity		
Antibody	Antigen	K_on_ (M^-1^ s^-1^)	K_off_ (s^-1^)	K_D_ (nM)	K_on_ (M^-1^ s^-1^)	K_off_ (s^-1^)	K_D_ (nM)
Ibalizumab	CD4	2.1 × 10^5^	1.8 × 10^−4^	0.8	5.7 × 10^5^	3.6 × 10^−5^	0.06
VκY91A	CD4	1.7 × 10^5^	4.2 × 10^−3^	25	ND^a^	ND	
VκR95A	CD4	3.0 × 10^5^	1.6 × 10^−2^	55	ND	ND	
V_H_Y99A	CD4	2.8 × 10^5^	2.0 × 10^−2^	72	ND	ND	
GA201	EGFR	2.5 × 10^5^	1.6 × 10^−4^	0.6	5.1 × 10^5^	2.3 × 10^−5^	0.05
VκF94A	EGFR	2.0 × 10^5^	1.4 × 10^−3^	7	ND	ND	
VκS93A+V_H_P97A	EGFR	1.4 × 10^5^	3.3 × 10^−3^	24	ND	ND	
VκF94A+V_H_P97A	EGFR	1.1 × 10^5^	1.6 × 10^−2^	148	ND	ND	
B1D2	HER2	3.1 × 10^5^	2.4 × 10^−4^	0.8	4.9 × 10^5^	4.4 × 10^−5^	0.09
H3B1	HER2	2.6 × 10^5^	4.8 × 10^−4^	1.8	ND	ND	
ML3-9	HER2	3.0 × 10^5^	2.7 × 10^−3^	9	ND	ND	
C6.5	HER2	3.8 × 10^5^	8.5 × 10^−3^	22	ND	ND	
G98A	HER2	3.3 × 10^5^	9.6 × 10^−2^	293	ND	ND	

^a^ND: not determined

Kinetic measurements to soluble monomeric forms of CD4, EGFR and HER2 were performed using an Octet384 instrument. The dissociation constants, K_D_, were calculated as the ratio of k_off_/k_on_ from a non-linear fit of the data

**Table 2:** Effector function activity of CD4 affinity-reduced IgG variants.

	ADCC			CDC			ADCP		
Antibody	EC_50_ (nM)	% cytotoxicity at max Ab conc.	*P*^a^	EC_50_ (nM)	% cytotoxicity at max Ab conc.	*P*	EC_50_ (nM)	% phagocytosis at max Ab conc.	*P*
Ibalizumab	0.2	57		n.a^b^	n.a		0.07	44	
VκY91A	0.6	63	<0.0001	34	27	0.0016	0.15	53	0.0027
VκR95A	1.1	77	<0.0001	7	83	<0.0001	0.22	57	0.0002
V_H_Y99A	0.8	84	<0.0001	16	98	<0.0001	0.14	59	<0.0001

^a^*P*: One way ANOVA for multiple comparisons was used to estimate statistically significant ADCC at max antibody concentration. Statistical significance was accepted for any P value < 0.05 at 95% confidence interval

^b^n.a: not available

### Generation and characterization of affinity-reduced anti-EGFR and anti-HER2 variants

To determine whether improved immune effector functions by modulation of antibody affinity holds true for diverse antibody sequences and target antigens, we generated an array of affinity-reduced variants of the anti-EGFR GA201 mAb [28] and also reconstructed the affinity-modulated variants of the anti-HER2 C6.5 mAb reported by Tang et al [26]. Based on homology modeling of the anti-EGFR GA201 variable domains using SAbDab software [29], we carried out alanine mutagenesis to exposed residues in CDRH3 and L3. We constructed 12 IgG variants carrying either a single mutation in CDRH3 or L3 or a combination of mutations in both CDRs and determined their binding kinetics to EGFR by Octet analysis. The intrinsic binding kinetics of three selected variants exhibiting a ~10-300-fold reduction in affinity compared with the parental sequence are shown (Table 1). Affinity-modulated anti-HER2 variants were constructed from synthetic genes based on the antibody sequences reported by Schier et al [30]. The antibodies were produced as full-length IgGs by mammalian expression and their intrinsic binding kinetics to HER2 were determined by Octet analysis (Table 1). The affinities reported by Tang et al [26] for three of the anti-HER2 variants, ML3-9 (7.3 nM), C6.5 (23 nM) and G98A (270 nM), are in good agreement with the kinetics we determined. However, the K_D_ values reported for the other two variants; H3B1 (0.56 nM) and B1D2 (0.028 nM), were ~3-fold lower than the values we measured (Table 1). Importantly, no significant change was observed between the association-rates (*K*_*on*_) of the affinity-modulated variants and their respective parental IgG, while the lower affinity variants of the three different mAbs exhibited approximately two-log reduction in dissociation-rates (*K*_*off*_) compared to their parental IgGs (Table 1). These kinetic properties assured that at any antibody concentration, the interaction of the affinity-modulated variants with the target cell is primarily affected by the differences in the *K*_*off*_. In addition, we confirmed the ability of the three high-affinity mAbs; ibalizumab, GA201 and B1D2 to cross-bind their target antigen in an avid manner by measuring their binding kinetics to biosensors coated with the target antigen. As shown in Table 1, the three mAbs displayed lower K_D_ values compared with the intrinsic K_D_ values reported in Table 1. These differences in K_D_ are primarily due to slower dissociation-rates resulting from avidity binding.

### Antibody affinity regulates the capacity to promote ADCC

**Fig 2:**
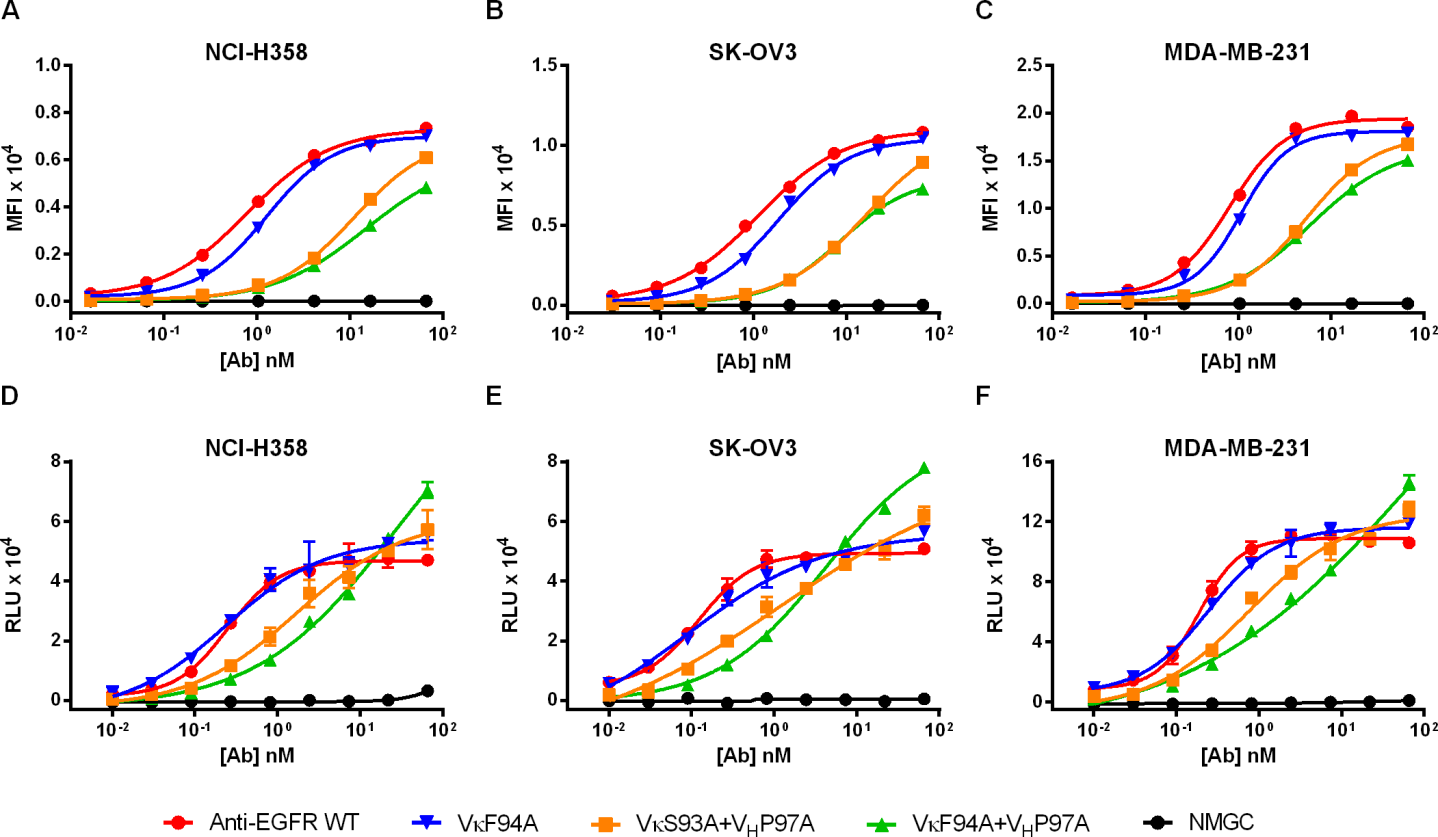
Cell binding and ADCC activity of anti-EGFR IgG variants. (A-C) Cell binding of anti-EGFR variants to NCI-H358, SK-OV3 and MDA-MB-231 cells, expressing; low, medium and high levels of EGFR, respectively. (D-F) ADCC activity of anti-EGFR variants against NCI-H358, SK-OV3 and MDA-MB-231 cells using NK92/NFAT cells as reporter cells. NMGC represents isotype control antibody. Each point represents the mean values of triplicate wells and the standard deviation is represented by error bars. See (Table 4) for statistical analysis and *P* values.

**Fig 3:**
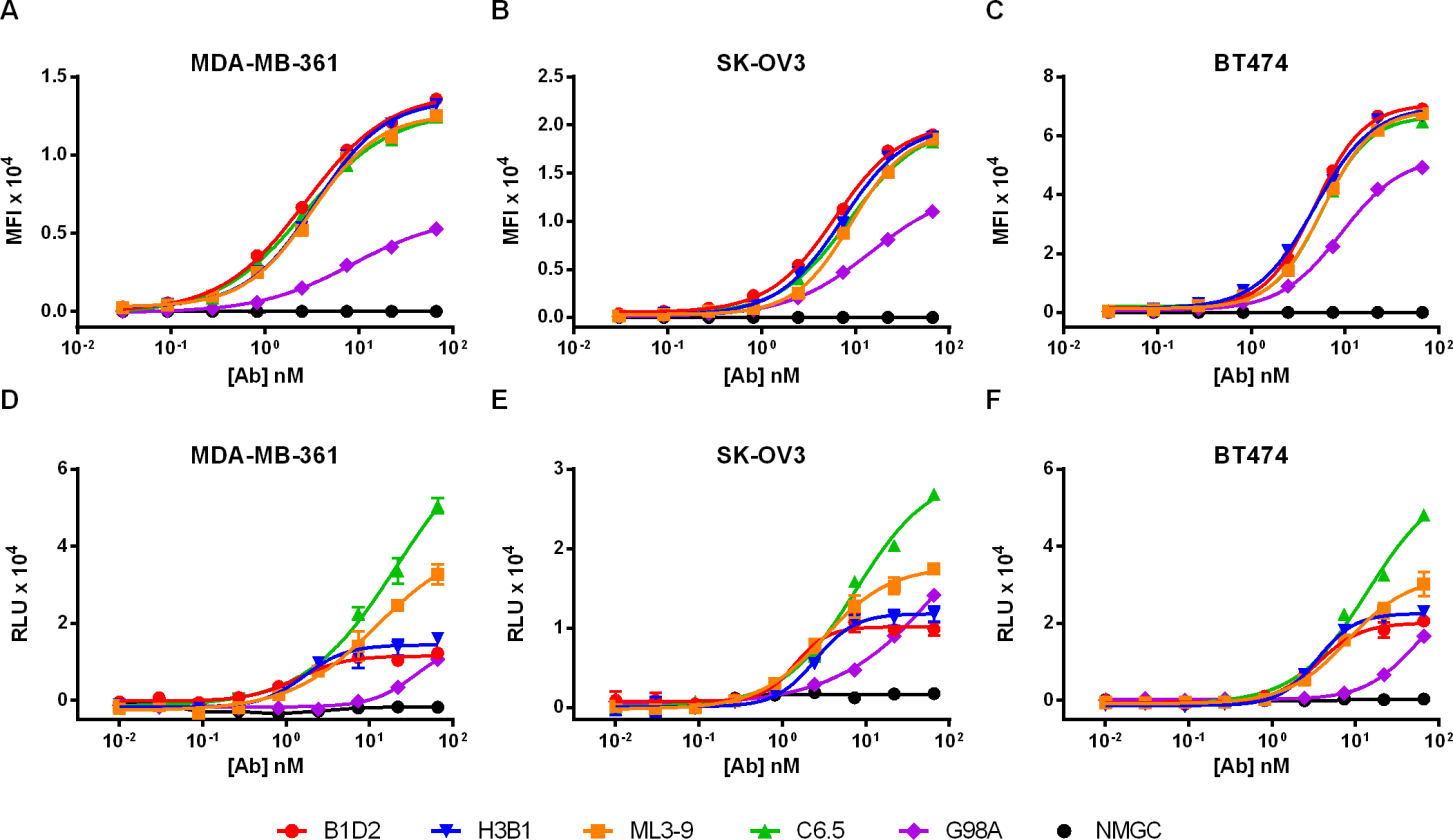
Cell binding and ADCC activity of anti-HER2 IgG variants. (A-C) Cell binding of anti-HER2 variants to MDA-MB-361, SK-OV3 and BT474 cells, expressing; low, medium and high levels of HER2, respectively. (D-F) ADCC activity of anti-HER2 variants against MDA-MB-361, SK-OV3 and BT474 cells using NK92/NFAT cells as reporter cells. NMGC represents isotype control antibody. Each point represents the mean values of triplicate wells and the standard deviation is represented by error bars. See (Table 5) for statistical analysis and *P* values.

To assess whether antibody affinity affects the capacity to promote effector functions, anti-EGFR and anti-HER2 variants were tested for their ability to mediate ADCC against a panel of tumor cell lines expressing diverse levels of EGFR and HER2 antigens. For each target antigen we selected three cell lines, expressing; low, medium and high levels of EGFR and HER2, respectively, as determined by receptor density analysis (Table 3). The IgG variants were first tested for cell binding to their respective target cells. As with the anti-CD4 variants, anti-EGFR (Fig 2A–2C) and anti-HER2 (Fig 3A–3C) variants with enhanced affinities exhibited either improved or similar cell binding properties compared with the low-affinity variants. For ADCC assessments we employed the highly sensitive NK92/NFAT reporter ADCC assay to allow high resolution between the tested variants. As shown, at saturating antibody concentrations, affinity-reduced anti-EGFR (Fig 2D–2F) and anti-HER2 (Fig 3D–3F) variants (with the exception of G98A) induced potentiated ADCC activity compared with the high-affinity mAbs. We speculate that the low-moderate cytotoxic activity induced by variant G98A is likely due to its exceptionally low-affinity (293 nM). Consistent with the results obtained with the anti-CD4 variants, the high-affinity anti-EGFR (Table 4) and anti-HER2 (Table 5) mAbs exhibited lower EC_50_ values compared with affinity-reduced variants, however, at max antibody concentrations, reduced target affinity correlated with superior reporter-based cytotoxicity. Again, the level of cytotoxic activity at max concentrations was inversely proportional to the reduced intrinsic affinity to the target antigen. Notably, the ability of both the anti-EGFR and anti-HER2 variants to mediate enhanced cytotoxic activity at saturating antibody concentrations was irrespective of the receptor density on targeted cells. Our preliminary data show that antibody intrinsic affinity to the target antigen clearly regulates the ability of affinity-modulated anti-EGFR and anti-HER2 antibodies to induce CDC in cancer cell lines previously shown to be resistant to complement-mediated attack (manuscript in preparation).

**Table 3:** EGFR and HER2 receptor density on human tumor cell lines.

Cell	EGFR	HER2
NCI-H358	1.0 × 10^4^	ND^a^
SK-OV3	5.3 × 10^4^	7.5 × 10^5^
MDA-MB-231	2.2 × 10^5^	ND
MDA-MB-361	ND	1.4 × 10^5^
BT474	ND	1.8 × 10^6^

^a^ND: not determined

**Table 4:** Reporter-based cytotoxicity of anti-EGFR IgG variants.

Cell	NCI-H358			SK-OV3			MDA-MB-231		
Antibody	EC_50_ (nM)	RLU^a^ at max Ab conc.	*P*^b^	EC_50_ (nM)	RLU at max Ab conc.	*P*	EC_50_ (nM)	RLU at max Ab conc.	*P*
GA201	0.25	47027		0.12	50813		0.18	106040	
VκF94A	0.24	55720	0.0051	0.10	56714	0.0006	0.25	118966	0.0018
VκS93A+V_H_P97A	1.72	57236	0.0041	1.27	62020	<0.0001	0.76	128280	<0.0001
VκF94A+V_H_P97A	25.54	70480	<0.0001	4.24	78113	<0.0001	99.69	146193	<0.0001

^a^RLU: Relative units

^b^*P*: One way ANOVA for multiple comparisons was used to determine statistically significant ADCC at max antibody concentration. Statistical significance was accepted for any P value < 0.05 at 95% confidence interval

**Table 5:** Reporter-based cytotoxicity of anti-HER2 IgG variants.

Cell	MDA-MB-361			SK-OV3			BT474		
Antibody	EC_50_ (nM)	RLU^a^ at max Ab conc.	*P*^b^	EC_50_ (nM)	RLU at max Ab conc.	*P*	EC_50_ (nM)	RLU at max Ab conc.	*P*
B1D2	1.37	12226		1.42	9879		3.55	20582	
H3B1	1.67	16026	0.0249	2.47	11773	0.0239	3.47	23112	ns
ML3-9	12.13	32740	<0.0001	3.12	17471	<0.0001	8.12	30156	0.0007
C6.5	21.30	50613	<0.0001	7.62	26854	<0.0001	13.81	48156	<0.0001
G98A	37.56	10646	ns^c^	111.73	14146	<0.0001	52.51	16635	ns

^a^RLU: Relative units

^b^*P*: One way ANOVA for multiple comparisons was used to determine statistically significant ADCC at max antibody concentration. Statistical significance was accepted for any P value < 0.05 at 95% confidence interval

^c^ns: not significant

**Fig 4:**
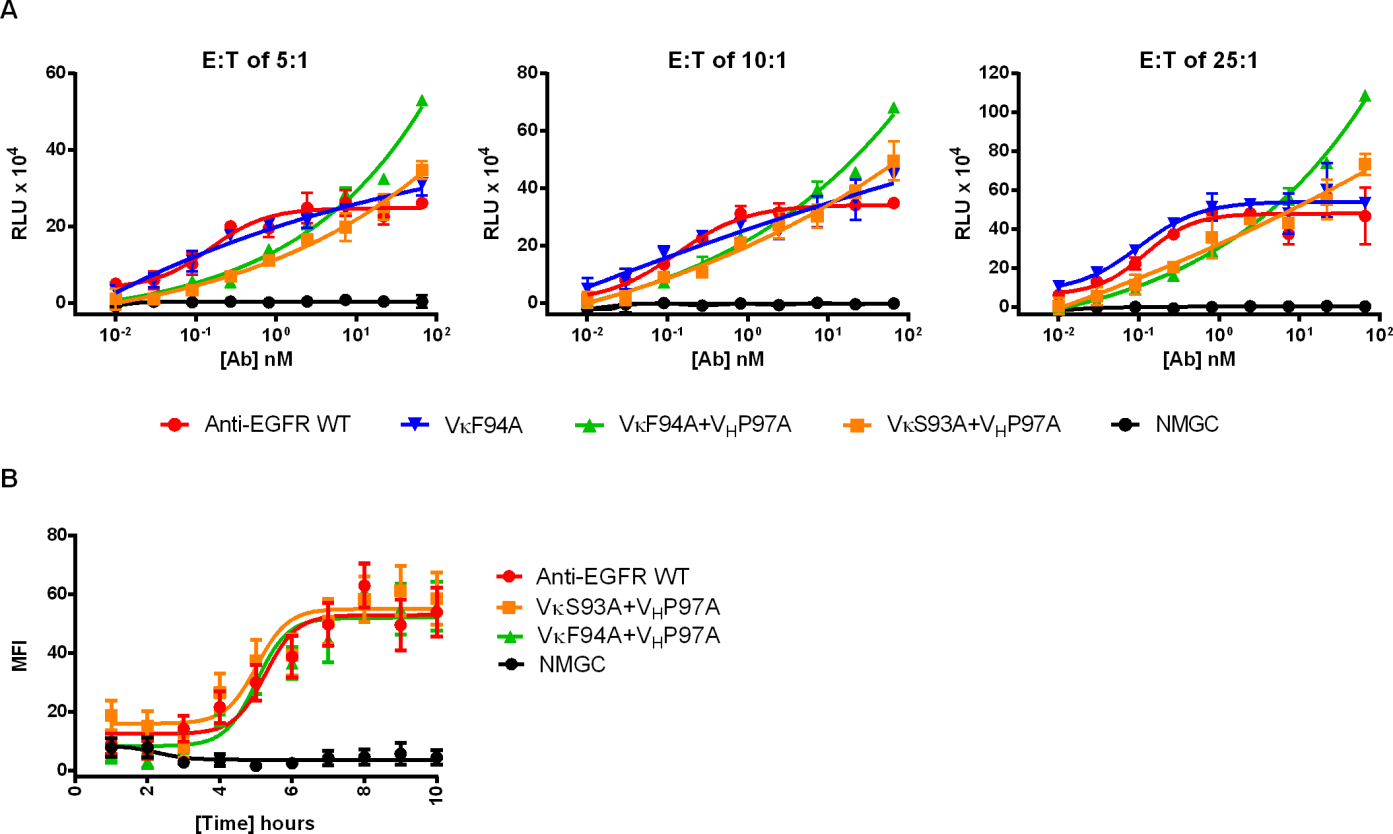
Effect of E:T ratios and cellular internalization on ADCC activity. (A) ADCC activity of anti-EGFR variants against SK-OV3 cells at varying E:T ratios. (B) Time course internalization of parental anti-EGFR and variants; VκS93A+V_H_P97A and VκF94A+V_H_P97A into MDA-MB-231 cells. NMGC represents isotype control antibody. Each point represents the mean values of triplicate wells and the standard deviation is represented by error bars.

To confirm that the enhanced ADCC observed with the affinity-reduced IgG variants was not affected by the effector to target (E:T) ratio, we compared the cytotoxic activity induced by the affinity-modulated anti-EGFR variants against SK-OV3 cells at E:T ratios of 5:1, 10:1 and 25:1. As shown in Fig 4A, the variants maintained their ADCC properties at all E:T ratios. To examine whether the reduced ADCC activity detected with the high-affinity variants was the result of enhanced cellular internalization leading to lower-density of antibody-Fc on the target cell, we compared the internalization rates of the parental anti-EGFR and variants, VκS93A+V_H_P97A and VκF94A+V_H_P97A, which represent high, moderate and low intrinsic affinity, respectively, into SK-OV3 cells. As shown in Fig 4B, all three antibodies exhibited similar internalization pattern at the exanimated time course. These results indicate that differences in the internalization properties of IgG variants with altered affinity was not the root for the reduced ADCC recorded for the high-affinity parental IgG. We further demonstrated that the binding affinity of the three anti-EGFR IgGs to the two isoforms of FcγRIIIA, high-affinity 158V and low-affinity 158F was indistinguishable as determined by steady-state equilibrium binding analysis on ProteOn (Table 6). Taken together, our data show that antibody intrinsic affinity to the target antigen clearly influenced the extent and efficiency of Fc-mediated effector functions.

**Table 6:** Equilibrium binding of anti-EGFR IgGs to FcγRIIIa isoforms.

	Ligand K_D_ (nM)	
Antibody	FcγRIIIa (158F)	FcγRIIIa (158V)
GA201	1620	165
VκS93A + V_H_P97A	1620	168
VκF94A + V_H_P97A	1640	168

Kinetic measurements to human FcγRIIIA isoforms were performed on a surface plasmon resonance-based ProteOn XPR36 biosensor. The dissociation constants, K_D_, were calculated from equilibrium binding

### Effect of binding valence to target antigen on effector function potency

**Fig 5:**
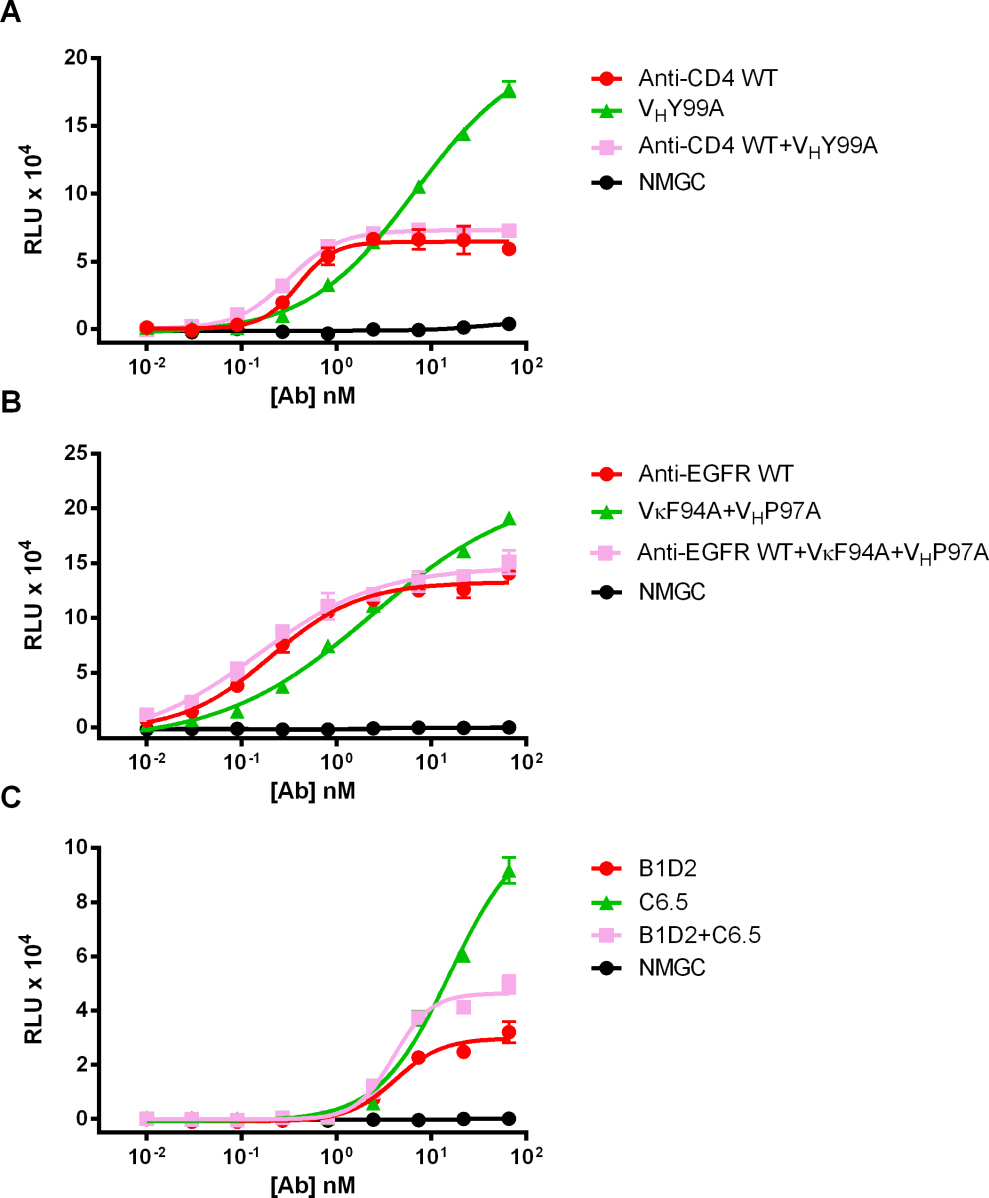
Competition ADCC studies. (A) ADCC activity of parental ibalizumab and anti-CD4 variant V_H_Y99A alone or in combination against CD4^+^ T cells. (B) ADCC activity of parental GA201 and anti-EGFR variant VκF94A+V_H_P97A alone or in combination against MDA-MB-231 cells. (C) ADCC activity of anti-HER2 B1D2 and C6.5 IgGs alone or in combination against BT474 cells. NMGC represents isotype control antibody. Each point represents the mean values of triplicate wells and the standard deviation is represented by error bars.

In an effort to understand how antibody binding affinity to the target antigen regulates effector function potency, we compared the levels of cytotoxicity mediated by the high-affinity mAbs and their respective affinity-reduced variants when tested alone or in combination. As shown in Fig 5, a 1:1 mixture of the high-affinity IgG with its affinity-reduced variant resulted in a significant reduction in ADCC activity compared to the activity of the affinity-reduced variant alone. These results suggest that a high-affinity antibody can compete and lessen the activity mediated by a lower affinity variant. We speculate that affinity-reduced variants with faster off-rates are likely to dissociate each binding arm from the cell surface more rapidly, resulting in a dominant monovalent binding mode. In contrast, high-affinity antibodies that display very slow off-rates induce a strong avidity effect and are likely to interact bivalently with the target cell.

**Fig 6:**
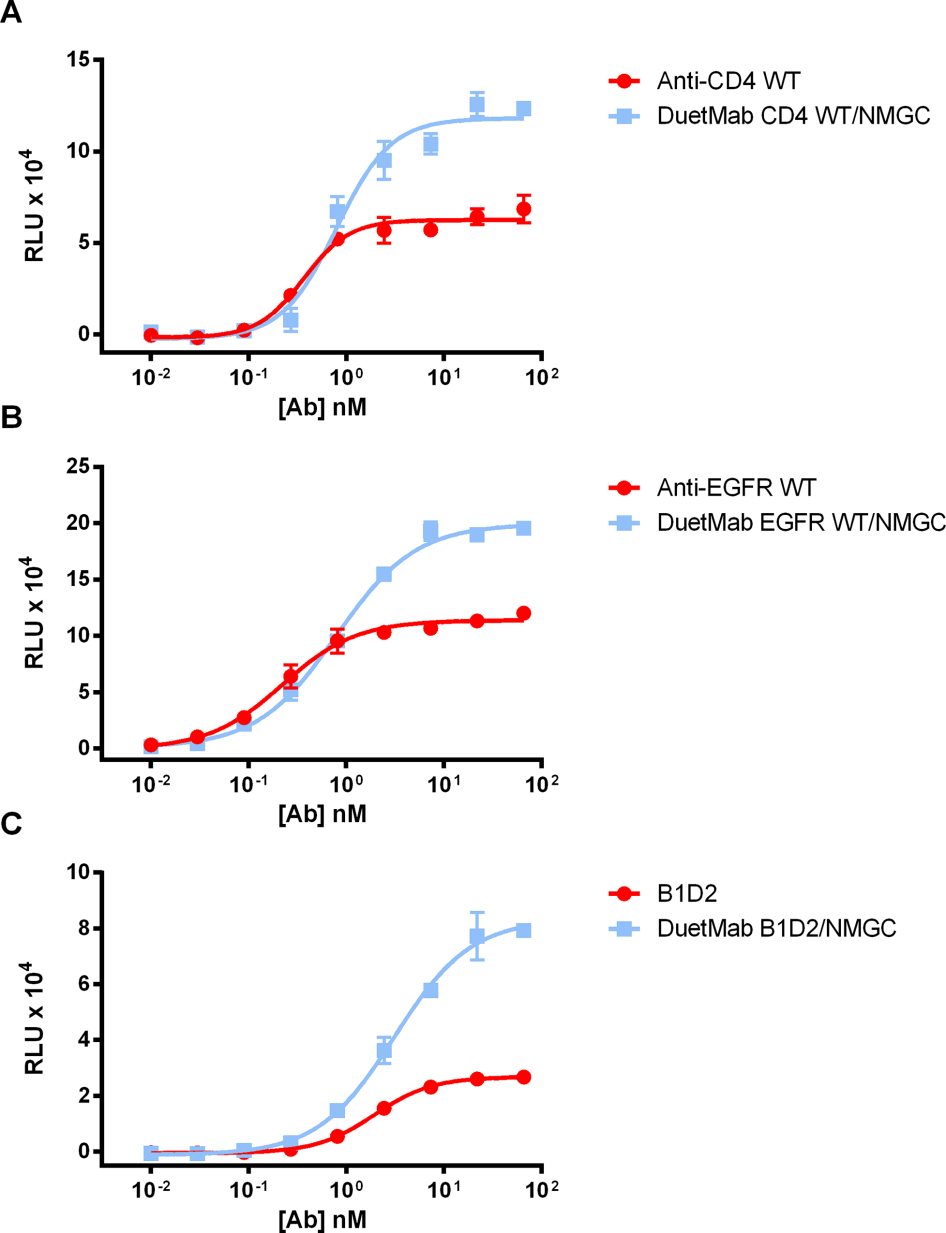
Effect of bivalent *vs*. monovalent antigen binding on ADCC activity. (**A**) ADCC activity of anti-CD4 ibalizumab formatted as either bivalent IgG or monovalent DuetMab against CD4^+^ T cells. (**B**) ADCC activity of anti-EGFR GA201formatted as either bivalent IgG or monovalent DuetMab against MDA-MB-231 cells. (**C**) ADCC activity of anti-HER2 B1D2 formatted as either bivalent IgG or monovalent DuetMab against BT474 cells. Each point represents the mean values of triplicate wells and the standard deviation is represented by error bars.

To elucidate the role of bivalent versus monovalent binding in the capacity of a mAb to promote immune effector functions, we generated monovalent formats of the high-affinity ibalizumab, GA201 and B1D2 IgGs, using our previously described DuetMab platform [31]. In this format, the fragment antibody binding (Fab) domain of the above IgGs was paired with a Fab of an isotype control (‘NMGC’) to form a heterodimer monovalent bispecific IgG. These DuetMab molecules were produced from mammalian cells and their homogeneity and purity were determined as previously described [31] (data not shown). We then compared the levels of cytotoxic activity mediated by the high affinity IgGs and their corresponding monovalent DuetMab molecules. As shown in Fig 6, in all three cases the DuetMab variants induced a much more potent ADCC compared to their bivalent IgG counterparts. Taken together, our results suggest that monovalent binding to a target antigen may increase the amount of antibody-Fc domains on the cell surface, leading to improved engagement with effector cells and therefore better immune response.

## Discussion

Fc-mediated immune effector functions play an essential role in the capacity of many therapeutic mAbs to eradicate cancer cells. The ability to recruit and activate Fc-dependent immune effector mechanisms, which, in turn, eliminate cancer cells, has focused considerable efforts into technologies that enhance Fc-mediated effector functions. However, these efforts have entirely relied on improving the affinity between the antibody-Fc region and activating FcγRs expressed on effector cells or to C1q. In this study, we show that antibody intrinsic affinity to the target antigen evidently influences the extent and efficiency of Fc-mediated effector mechanisms. Using an array of affinity-modulated variants of three different mAbs, anti-CD4 ibalizumab, anti-EGFR GA201 and anti-HER2 C6.5, we have shown that at saturating antibody concentrations, 1) reduced binding affinity translates to enhanced effector functions, however, a threshold of minimum affinity is required; 2) the ability to mediate enhanced cytotoxic activity is irrespective of receptor density on targeted cells; and 3) antibody binding valence to the target antigen clearly regulates the extent and efficiency of effector functions. At the same time, antibodies with improved binding affinities to the target antigen consistently exhibited lower EC_50_ values compared with the corresponding low-affinity variants. Taken together, our findings may question the need for engineering antibody therapeutics with pM-fM affinities and may provide insights as to why antibodies generated by a natural humoral response, largely possess low-to-mid double digit nM affinities. Our results in part are in discrepancy with the data reported by Tang et al [26], even though the same antibody sequences and target cells were used in both studies. While in both studies enhanced binding affinity correlated with lower EC_50_ values, at saturating antibody concentrations of 10 μg/mL opposite results are reported. A possible explanation for the disparity between the two studies may be related to the purity of the antibodies tested. Effector function assays are highly sensitive to high molecular weight protein aggregates that due to enhanced avidity may confound the sensitivity and accuracy of the assays. We therefore rigorously purified the mAbs tested in this study from residual impurities that may influence the integrity of the effector function assays. Such rigorous purification process is not reported in the work of Tang et al [26].

**Fig 7:**
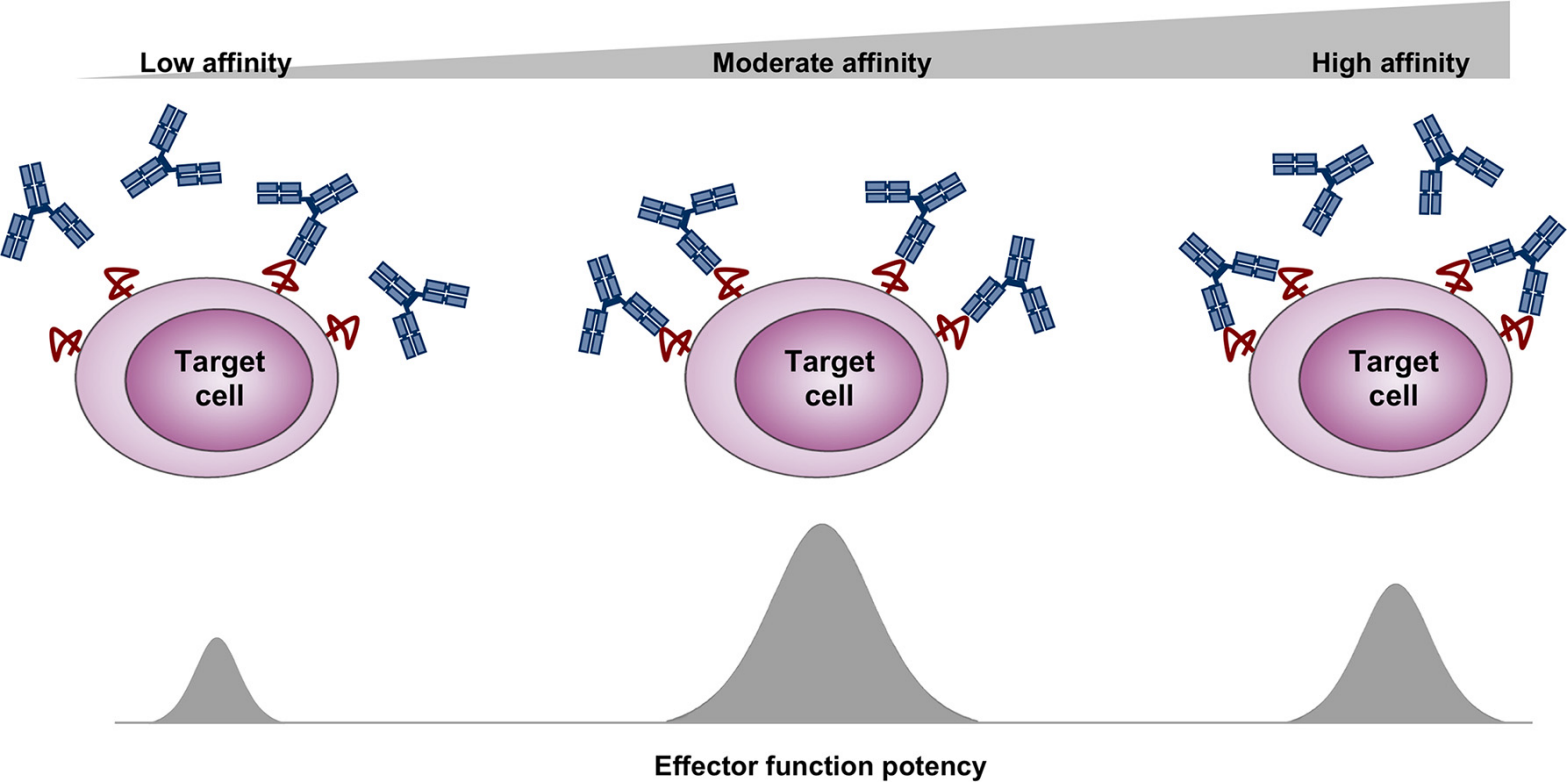
Proposed model for how antibody binding affinity to target antigen regulates effector function potency. Enhanced intrinsic affinity increases the likelihood for bivalent interaction with target cell. In contrast, a reduction in intrinsic affinity improves the probability for monovalent binding, leading to increased amount of antibody-Fc domains interacting with the target cell and hence better engagement with effector elements. However, additional reduction in intrinsic affinity, beyond a threshold of minimum affinity will result in poor cellular interaction and reduced effector functions.

To explain our findings, we propose the model illustrated in Fig 7. We speculate that high-affinity antibodies with slow off-rates that are capable of bridging two antigen molecules on the surface of the cell are likely to interact bivalently with the target cell due to strong avidity effect. In contrast, antibodies exhibiting faster off-rates are likely to dissociate each binding arm from the cell surface more rapidly, resulting in a higher likelihood of monovalent binding with the target cell. We therefore speculate that, at saturating antibody concentrations, monovalent binding will allow for more antibody molecules to interact with the target cell, resulting in increased amount of antibody-Fc domains decorating the cell surface. This should facilitate improved recruitment of effector elements and yield potentiated activity. However, in cases where antibody binding affinity is too low (very fast off-rates such as those of G98A), at equilibrium, not enough antibody molecules will be associated with the target cell to facilitate engagement with effector cells, resulting in an overall reduced effector activity.

As for the therapeutic relevance of saturating antibody concentrations in vivo, depending on the translational pharmacokinetics/pharmacodynamics (PK/PD) framework, the efficacious dose of most antibody therapeutics is determined based on 90% target suppression, provided no apparent dose-limiting toxicity [32]. Saturating concentrations are readily feasible in hematological malignancies, however are somewhat more challenging to attain in the case of solid tumors due to various factors that affect antibody distribution, such as vascularization, extravasation, interstitial diffusion or antibody catabolism at the tumor site. These determinants are largely overcome by repeated drug administration regimens that maintain an elevated plasma level over an extended period of time and eventually results in saturation of all binding sites within the tumor [33, 34].

In the near future, cancer treatment strategies will inevitably include immunotherapeutics that enhance ADCC, ADCP or CDC. Furthermore, a growing body of evidence suggests that anti-tumor antibodies mediating ADCC and ADCP activities may also stimulate vaccinal effect and long-term cellular immunity against cancer [35–40]. In addition, several emerging immunomodulatory antibodies, initially thought to mediate their activities primarily by antagonizing co-inhibitory checkpoints (CTLA-4) or agonizing co-stimulatory pathways (OX40 and GITR), may in fact be simply mediating depletion of regulatory T cells (Tregs) at the tumor site by inducing ADCC and ADCP [41–44]. For these reasons, a deeper understanding of the factors that regulate the capacity of mAbs to recruit and activate Fc-dependent immune effector mechanisms is imperative for the development of next-generation immunotherapeutics that augment effector functions. We believe that the findings we have identified in this study are key design parameters and should be taken into consideration when generating clinically relevant mAbs. Experiments are in progress to determine the in vivo relevance of these observations.

## Materials and Methods

### Data

All experiments depicted in this work are representative of at least 2 independent measurements.

### Statistical Analysis

Statistical analysis and plots were done using Graph Pad Prism software. For comparisons of multiple parametric variables we used One way ANOVA followed by Holm-Sidak’s multiple comparisons test. Statistical significance was accepted for any *P* value < 0.05 at 95% confidence interval.

### Cells

Human CD4^+^ T lymphocytes were obtained from PMBC of healthy donors as previously described [27]. Briefly, cells were selected by magnetic bead separation (Stemcell technologies) as per the manufacturer’s instructions and cultured in X-VIVO 15 chemically defined, serum-free medium with gentamicin and phenol red (Lonza) supplemented with 50 μM 2-mercaptoethanol (Gibco) and GlutaMAX (Gibco). Human tumor cell lines, NCI-H358, MDA-MB-231, MDA-MB-361, BT474 and SK-OV3, were obtained from the American Type Culture Collection (ATCC). NCI-H358, MDA-MB-231 and BT474 cells were cultured in RPMI-1640 with GlutaMAX supplemented with 10% HI FBS. MDA-MB-361 cells were cultured in Leitovitz’s medium (Gibco) supplemented with 20% HI FBS. SK-OV3 cells were cultured in McCoy’s medium (Gibco) supplemented with 10% HI FBS. The human KC1333 NK cell line (BioWa Potelligent Technology) stably expressing human FcγRIIIA and FcεRIγ was maintained in Advanced RPMI 1640 supplemented with 10% HI FBS, 200 μg/mL geneticin, 4 mM glutamine and 4.65 × 10^5^ IU/mL IL2. The NK92/NFAT cell line expressing the high-affinity FcγRIIIa-V158 receptor and a luciferase reporter gene driven by the NFAT promoter were generated as previously described [27]. These cells were cultured in RPMI-1640 supplemented with 12.5% HI FBS, 12.5% HI horse serum, 2 mM glutamine, 500 μg/mL geneticin, 100 μM 2-mercaptoethanol and 3.72 × 10^3^ IU/ml IL2. Macrophages were differentiated from the human monocytoid cell line THP-1 (ATCC) by addition of vitamin-D3 (Sigma) to a final concentration of 200 nM in RPMI 1640 with GlutaMAX supplemented with 10% HI-FBS and incubation for 4 days at 37°C in 5% CO_2_.

### Antibody mutagenesis and production

Alanine mutagenesis of targeted residues in CDRH3 and L3 of the anti-EGFR GA201 mAb was performed by site-directed mutagenesis using standard PCR techniques. The mutated VH and VL DNA fragments were cloned into an Orip/EBNA-1-based episomal mammalian expression plasmid, pOE [45] for production as human IgG1. For construction of affinity-modulated anti-HER2 antibodies, synthetic VH and VL genes of the corresponding antibody sequences were ordered from Integrated DNA Technology (IDT) and cloned into pOE plasmid. Production of human IgG1 and DuetMab antibodies was performed essentially as described [27]. Briefly, antibodies were transiently expressed in HEK293F cells using 293fectin™ (Invitrogen) and grown in serum-free Freestyle™ medium (Invitrogen) in accordance with the manufacturer's recommended procedures. Culture supernatants were collected 6 days after transfection and filtered through a 0.22 μm sterile filter. Antibody concentration in cell culture supernatants was determined using an Octet384 instrument (ForteBio) according to the supplier’s protocol. Antibodies were purified by affinity chromatography on a protein A column using MabSelect SuRe resin (GE Healthcare) and subsequently buffer-exchanged in phosphate buffered saline (PBS) pH 7.2. Aggregated protein was separated from monomeric antibodies by size exclusion chromatography using a Superdex 200 column (GE Healthcare). Monomeric antibody fractions were pooled and stored as 1.0 mg/mL aliquots at -80°C. The concentration of purified antibodies was determined by their absorbance at 280 nm.

### Binding kinetics measurements

Kinetic measurements to soluble monomeric forms of CD4 (R&D Systems), EGFR (R&D Systems) and HER2 (eBioscience) ligands were measured by biolayer interferometry on an Octet384 instrument (ForteBio) essentially as described [27]. Briefly, for assessmnet of intrinsic binding affinity, antibodies at 10 μg/mL in PBS pH 7.2, 3 mg/mL BSA, 0.05% (v/v) tween 20 (assay buffer) were captured on anti-human IgG Fc biosensors (ForteBio). The loaded biosensors were washed with assay buffer to remove any unbound protein before measuring association and dissociation with serial dilutions of the antigen ligands. For determination of apparent binding affinity, streptavidin biosensors (ForteBio) were loaded with biotinylated CD4, EGFR or HER2 antigens at 5 μg/mL in assay buffer. Following washing, association and dissociation meaurments were carried out using serial dilutions of the purified IgGs. The dissociation constant (K_D_), was deduced as the ratio of the two rate constants (k_off_/k_on_) from a non-linear fit of the data using the Octet384 software v.7.2.

Kinetic measurements to recombinant human FcγRIIIA isoforms were performed on a surface plasmon resonance-based ProteOn XPR36 array system (Bio-Rad). Antibodies at 50 μg/mL were immobilized on a GLC sensor chip using a ProteOn^TM^ amine coupling kit (Bio-Rad) according to the manufacturer’s instructions. Excess reactive groups were blocked with 1 M ethanolamine. Serial dilutions of FcγRIIIA isoforms in PBS, pH 7.4, 0.005% Tween 20 (v/v), 3 mM EDTA were passed over the immobilized surface. Equilibrium dissociation constants (K_D_) were calculated from equilibrium binding rates using the ProteOn^TM^ Manager software.

### IgG cell binding assays

Cellular binding studies were performed by flow cytometry using a LSR II (Becton Dickinson) instrument essentially as described [27]. ~ 5 × 10^4^ cells/well were used in each experiment. Cells were washed twice with PBS pH 7.2, 2% FBS, 2 mM EDTA and 0.1% sodium azide (FACS buffer) and incubated with serial dilutions of the tested antibodies for 1 h at 4°C. Following a washing step with FACS buffer, FITC-conjugated goat anti-human Fcγ (Jackson ImmunoResearch) was added for 45 min at 4°C. Analysis was conducted with FlowJo software (Tree Star), and the mean fluorescence intensity (MFI) was used to determine the amount of IgGs bound on the cell surface.

### Receptor density analysis

Receptor density studies were performed by flow cytometry on a MACSQuant VYB (Miltenyl Biotec) essentially as described [27]. Briefly, anti-CD4 (ibalizumab), anti-EGFR (GA201) and anti-HER2 (B1D2) IgGs were first labeled with Alexa Fluor 647 labeling kit (Invitrogen) according to the manufacturer’s instructions. Antibody concentration and fluorochrome to protein (F:P) ratio were calculated by a ND-1000 spectrophotomer (NanoDrop). Cells at ~ 4 × 10^6^ cells/mL were first washed with ice-cold FACS Buffer (PBS pH 7.2, 2% FBS, 2 mM EDTA and 0.1% sodium azide) followed by incubation with saturating concentration (≥ 20 μg/mL) of conjugated antibodies for 30 min at 4°C. After washing with FACS buffer, cells were fixed in ice-cold 1.8% paraformaldehyde (PFA) and detection of bound antibodies was determined on MACSQuant VYB using MACSQuantify™ software. Data were analyzed with the FlowJo analysis software. For quantitation of CD4, EGFR and HER2 density on cells, Quantum Alexa Fluor 647 MESF (Molecules of Equivalent Soluble Fluorochrome) beads (Bangs Laboratories) were processed on the flow cytometer using similar settings. QuickCal program (Bangs Laboratories) was used to establish a standard curve. The calculated MESF was then divided by the antibody F:P ratio to give a corrected Antibody Binding Capacity (ABC).

### Antibody internalization analysis

Antibody internalization was determined by live cell imaging using a Cellomics Arrayscan VTI (ThermoFisher Scientific). Antibodies were first chemically conjugated with pHAb dye (Promega) on Magne™ Protein A beads (Promega), according to the manufacturer’s instructions. Antibody concentration and dye to antibody ratio (DAR) of recovered antibody was calculated using a ND-1000 spectrophotomer (NanoDrop). To enable cell identification and imaging, MDA-MB-231 cells were transduced with lentiviral vector carrying the GFP gene. MDA-MB-231-GFP cells in RPMI 1640 with GlutaMAX supplemented with 10% FBS were seeded into 96-well at a density of ~ 2 × 10^4^ cells/well and allowed to adhere overnight. The following day pHAb-antibodies at 10 μg/mL were added to the cells in fresh media with no phenol red and antibody internalization was measured by high content screening on a Cellomics Arrayscan VTI. Live cells were imaged over 10 hours and data were captured every 20 minutes. The degree of co-localization of the pHAb-antibodies with individual cells, identified by GFP signal, was quantitated using the co-localization bio-application in HCS Studio V2 software (ThermoFisher Scientific).

### Antibody-dependent cell-mediated cytotoxicity (ADCC) assays

ADCC activities were measured by flow-based cell enumeration method essentially as described [27]. Briefly, target cells were initially stained with CellTrace^TM^ CFSE dye (Invitrogen) to enable subsequent identification by flow cytometry. KC1333 NK effector cells at an effector:target (E:T) ratio of 2.5:1 were added in the presence or absence of various concentrations of antibodies and the culture was incubated for 6 h at 37°C in 5% CO_2._ Next, cells were stained with propidium iodide (PI) and PE-Cy7 CD16 and analyzed by flow cytometry on an LSR Fortessa (Becton Dickinson). To enumerate target cell populations, data were processed using the FlowJo analysis software. Live cells were separated by tracer dye and counted within a defined time gate. Cytotoxicity was determined by measuring the change in cell quantity relative to a no-antibody control. For high-resolution ADCC analysis we used the NK92/NFAT reporter assay which relied on a bioluminescent marker to quantify functional ADCC. This assay was performed essentially as we previously described [27]. Briefly, ADCC activity was extrapolated from the binding mediated by effector NK92/NFAT cells, stably expressing the high-affinity FcγRIIIa-V158 receptor and a luciferase/NFAT response element to cell-bound antibody. Target cells were seeded in 96-well plates at a density of ~ 1 × 10^4^ cells/well in RPMI 1640 with GlutaMAX and supplemented with 12.5% HI FBS, 12.5% HI horse serum, 500 μg/mL geneticin, and 100 μM 2-mercaptoethanol. NK92/NFAT cells were added at E:T ratios varying from 1:1 to 25:1 in the presence or absence of various concentrations of antibodies and the culture was incubated for 5 h at 37°C in 5% CO_2_. After treatment, the cells were exposed to Steady-Glo luciferase substrate (Promega) for ~ 50 min and OD409 was measured using an EnVision 2104 Multilabel plate reader (PerkinElmer).

### Complement-dependent cytotoxicity (CDC) assays

CDC assays were performed using a Celigo Imaging Cytometer (Nexelcom). Target cells were stained with Calcein AM dye (ThermoFisher) according to the manufacturer’s instructions. Cells were then seeded in 96-well plates at a density of ~ 1 × 10^4^ cells/well in RPMI 1640 with GlutaMAX supplemented with 5% FBS. Complement (baby rabbit complement; Cedarlane) was then added to a final concentration of 11% and cells were incubated for 3 h at 37°C in 5% CO_2_. Calcein AM positive cells were then quantified using Celigo software. Six wells per plate without added antibodies (but with complement) served as the “no specific lysis” control wells. Specific lysis (% cytotoxicity) was calculated as 100 − ((# of Calcein AM positive cells in test well)/(mean # of Calcein AM cells in no antibody control wells) × 100).

### Antibody-dependent cell-mediated phagocytosis (ADCP) assays

ADCP activities were measured by flow-based quantification method similar to that we previously described [46]. Briefly, differentiated macrophages and target human CD4^+^ T cells were labeled with CellTrace^TM^ Violet and CFSE (Invitrogen), respectively, according to the manufacturer’s instructions. Target cells were then seeded in 96-well plates at a density of ~ 1 × 10^4^ cells/well in RPMI 1640 with GlutaMAX supplemented with 10% HI FBS and incubated with macrophage effector cells at an E:T ratio of 4:1. Antibodies at various concentrations were added and the cells were incubated for 2 h at 37°C in 5% CO_2_. Cells were then stained with Live/Dead Far Red (ThermoFisher) and analyzed by flow cytometry on an LSR II (BD Biosciences). To determine cell phagocytosis, data were processed using the FlowJo analysis software. Data were gated on single, live cells, and % phagocytosis was calculated as: 100 × (count CFSE^+^, CellTrace Violet^+^ cells)/(total count CFSE^+^ cells).

## Abbreviations

IgG: Immunoglobulin G
mAbs: monoclonal antibodies
CDR: complementarity-determining region
Fc: fragment crystallizable
Fab: fragment antibody binding
FcγR: Fc-gamma receptors
C1q: complement 1q
ADCC: antibody-dependent cell-mediated cytotoxicity
CDC: complement-dependent cytotoxicity
ADCP: antibody dependent cell-mediated phagocytosis
E:T: ratio of effector to target cells
K_D_: dissociation constant
PBS: phosphate buffered saline
